# Multifunctional Hydrogels Based on Cellulose and Modified Lignin for Advanced Wounds Management

**DOI:** 10.3390/pharmaceutics15112588

**Published:** 2023-11-04

**Authors:** Diana Elena Ciolacu, Raluca Nicu, Dana Mihaela Suflet, Daniela Rusu, Raluca Nicoleta Darie-Nita, Natalia Simionescu, Georgeta Cazacu, Florin Ciolacu

**Affiliations:** 1 Department of Natural Polymers, Bioactive and Biocompatible Materials, “Petru Poni” Institute of Macromolecular Chemistry, 700487 Iasi, Romania; nicu.raluca@icmpp.ro (R.N.); dsuflet@icmpp.ro (D.M.S.); 2 Department of Physics of Polymers and Polymeric Materials, “Petru Poni” Institute of Macromolecular Chemistry, 700487 Iasi, Romania; rusu.daniela@icmpp.ro; 3 Department of Physical Chemistry of Polymers, “Petru Poni” Institute of Macromolecular Chemistry, 700487 Iasi, Romania; darier@icmpp.ro (R.N.D.-N.); gcazacu@icmpp.ro (G.C.); 4 Center of Advanced Research in Bionanoconjugates and Biopolymers, “Petru Poni” Institute of Macromolecular Chemistry, 700487 Iasi, Romania; natalia.simionescu@icmpp.ro; 5 Department of Natural and Synthetic Polymers, “Gheorghe Asachi” Technical University of Iasi, 700050 Iasi, Romania

**Keywords:** cellulose, modified lignin, hydrogel, controlled drug release, antibacterial activity, biocompatibility, wound dressing

## Abstract

Considering the complex process of wound healing, it is expected that an optimal wound dressing should be able to overcome the multiple obstacles that can be encountered in the wound healing process. An ideal dressing should be biocompatible, biodegradable and able to maintain moisture, as well as allow the removal of exudate, have antibacterial properties, protect the wound from pathogens and promote wound healing. Starting from this desideratum, we intended to design a multifunctional hydrogel that would present good biocompatibility, the ability to provide a favorable environment for wound healing, antibacterial properties, and also, the capacity to release drugs in a controlled manner. In the preparation of hydrogels, two natural polymers were used, cellulose (C) and chemically modified lignin (LE), which were chemically cross-linked in the presence of epichlorohydrin. The structural and morphological characterization of CLE hydrogels was performed by ATR-FTIR spectroscopy and scanning electron microscopy (SEM), respectively. In addition, the degree of swelling of CLE hydrogels, the incorporation/release kinetics of procaine hydrochloride (PrHy), and their cytotoxicity and antibacterial properties were investigated. The rheological characterization, mechanical properties and mucoadhesion assessment completed the study of CLE hydrogels. The obtained results show that CLE hydrogels have an increased degree of swelling compared to cellulose-based hydrogel, a better capacity to encapsulate PrHy and to control the release of the drug, as well as antibacterial properties and improved mucoadhesion. All these characteristics highlight that the addition of LE to the cellulose matrix has a positive impact on the properties of CLE hydrogels, confirming that these hydrogels can be considered as potential candidates for applications as oral wound dressings.

## 1. Introduction

Wound healing is a complicated process that is affected by local and systemic factors, requiring a longer period of time to reach maximum healing capacity and an appropriate environment [1]. Wound management must take into account the wound complexity and collaborative processes, which include wound pre-cleaning, wound healing, infection prevention, germ treatment and the treatment strategy [2,3]. The advanced wound management strategies must achieve the non-invasive monitoring of healing, manage pain and allow the controlled release of substances capable of promoting wound regeneration [4]. 

Medical dressings are essential devices in healthcare and play critical roles in wound treatments. A dressing that can be used for wound healing must meet several conditions, including maintaining moisture on the wound surface, protecting the wound from infection, absorbing exudates, and reducing pain in the wounds [5,6]. Furthermore, an oral wound dressing must overcome all the challenges brought by the oral environment, which consist of (i) changes in temperature and pH caused by the presence of saliva and (ii) the formation of biofilms due to the presence of different types of bacteria [7]. In order to fulfill all these goals, different multifunctional wound dressing materials have been designed, which are able to act at the same time on various aspects of the wound healing process. Moreover, wound dressing materials that would present a reasonable cost, fast-healing capacity and minimal inconvenience for the patients have been sought. In recent years, numerous wound dressings with excellent properties, such as antibacterial properties and self-healing ability, have been successfully developed to speed up wound healing [8]. Among these biomaterials, hydrogels are the most promising interactive dressings due to their active intervention in the wound healing process, through the controlled delivery of drugs, growth factors or other therapeutic agents. Hydrogels present essential characteristics in promoting wound healing, such as: (i) a calming effect—due to their moisture content, an optimal microclimate develops between the wound bed and the dressing; (ii) they reduce the pain associated with dressing changes; (iii) their limited adhesion allows for easy removal from the wound without causing additional trauma to the healing tissue; (iv) their transparent nature allows for the clinical assessment of the healing process without the removal of the dressing [4]. Hydrogels based on natural polymers have gained attention due to their significant potential in the regeneration process, highlighted also by the possibility of encapsulating active substances that give them antibacterial, anti-inflammatory and antioxidant properties.

A cellulose-based hydrogel has water-absorbing properties; in this particular case, it can absorb tissue exudates, contribute to the removal of bacteria from wounds and provide excellent moisture conditions for the wound healing process [9]. Many studies have focused on the use of cellulose in the development of wound dressings, due to its excellent properties, such as: biocompatibility, biodegradability, non-toxicity, hydrophilicity, abundance and cost-effectiveness. Cellulose derivatives have been the most widely studied for this purpose, as they have suitable applicability to the development of hydrogel-based dressings that can contribute to wound healing [10]. Cellulose can be used in combination with different natural or synthetic polymers to obtain hydrogels with three-dimensional (3D) networks, presenting improved properties and combining the performance of each individual component. This is the case for hydrogels prepared from cellulose and linseed gum, wherein it was confirmed that the presence of linseed gum improves the mechanical properties of hydrogels and their thermal stability (TG and DSC) [11]. Moreover, an example where the presence of cellulose contributes to improving the mechanical properties of hydrogels is hydrogels based on cellulose and dextran [12]. These hydrogels have demonstrated their ability to facilitate and accelerate wound healing by inhibiting the inflammation process through the controlled and sustained release of the anti-inflammatory bioactive compounds (polyphenols, PFs). 

Lignin, considered the second most abundant biopolymer, is an attractive candidate in the preparation of hydrogels used in biomedical applications. Due to its antibacterial and antioxidant properties, lignin has drawn increased attention for its application in wound healing [13]. Lignin used in combination with natural (chitosan, gelatin, agarose, carrageenan) or synthetic (polyvinyl alcohol) polymers in hydrogel preparation has provided new opportunities for effective wound care and management [14,15,16]. It was shown that the introduction of lignin into cellulose-based hydrogels effectively improved the adsorption capacity and contributed to the controlled release of drugs (polyphenols, PFs) [17]. Hydrogels made from bacterial cellulose and lignin enzymatically synthesized from coniferyl alcohol were designed to have an inhibitory/bactericidal effect against clinically isolated biofilm-forming bacteria (*P. aeruginosa*, *S. aureus* and *Serratia* sp.) and laboratory strains (*S. aureus*, *L. monocytogenes* and *S. typhimurium*), and to be used in wound healing [18]. 

In this context, this study aims to report the preparation and characterization of new multifunctional hydrogels based on cellulose and lignin chemically modified with epoxy groups. To our knowledge, there are no up-to-date studies reporting the preparation of this type of bicomponent hydrogel. The expected outcome of this study is to obtain improved properties in these hydrogels compared to cellulose–lignin hydrogels from our previous work [17], in terms of their swelling capacity and controlled drug release. Moreover, the multifunctional hydrogels thus obtained are intended to have the ability to reduce pain by releasing procaine in a controlled manner, antibacterial properties induced by the components of the 3D network, and biocompatibility, all these with a view to their possible application in oral wound management.

## 2. Materials and Methods

### 2.1. Materials

Microcrystalline cellulose (C) was purchased from Sigma-Aldrich (Avicel PH-101, DP = 180; Sigma-Aldrich GmbH, Darmstadt, Germany). 

The chemical modification of steam explosion lignin (L; produced by the steam explosion of aspen wood (*Populus tremuloides*); ENEA, Italy) was achieved in the presence of 20% NaOH solution, with epichlorohydrin (ECH) at a molar ratio of L:ECH = 1:10, for 5 h at 75 °C. Two fractions of modified lignin were obtained: a solid fraction, which was separated from the mixture by filtration, and a liquid fraction, which was dissolved in absolute methanol, filtrated and then concentrated. This concentrated fraction of chemically modified lignin (LE) has a brown color, high viscosity, and is soluble in water and methanol. The obtained LE has an epoxy equivalent of 0.2% and a dynamic viscosity of 18,000 cP.

Epichlorohydrin (ECH) was purchased from Sigma-Aldrich (purity > 99 %; d = 1.18 g/cm^3^; Darmstadt, Germany) and was used without further purification. Sodium hydroxide (NaOH) in pellets (purity ≥ 97%) was supplied by Merck (Merck KGaA, Darmstadt, Germany). Procaine hydrochloride (PrHy), with m.p. 155–156 °C and purity ≥ 97%, was also supplied by Sigma-Aldrich (Darmstadt, Germany). 

#### 2.1.1. Preparation of CLE Hydrogels

CLE hydrogels were prepared in different gravimetric ratios of cellulose (C) and modified lignin (LE), through the following procedure: 0.5 g polymer was added into a 6.7 mL 8.5% NaOH solution and frozen at low temperature (−30 °C). After thawing, 2.14 mL of ECH was added under continuous stirring. The obtained composition was maintained for 5 h at 80 °C. The hydrogels were washed with warm distilled water (60 °C) for 15 days in order to remove the excess NaOH, any ECH traces, and NaCl, followed by lyophilization. 

#### 2.1.2. Preparation of PrHy-Loaded CLE Hydrogels

CLE hydrogels were immersed in 50 mL of 4 g/L PrHy solution for 72 h at room temperature to allow the PrHy to diffuse into the hydrogels, and finally lyophilized in a freeze dryer ALPHA 1-2/LD (Martin Christ Drying Systems GmbH, Osterode, Germany) for 48 h. CLE hydrogels loaded with PrHy are denoted as P-CLE.

### 2.2. Methods

#### 2.2.1. Swelling Measurements

Swelling studies of CLE hydrogels were performed in distilled water, at 37 °C. The samples were periodically taken out and gently wiped with a soft tissue to remove the excess water, weighed, and then returned into the vessel. The swelling degree (*Q_max_*, %) of the hydrogels was calculated according to Equation (1):

(1) $$Q_{max}=\frac{M_{s}-M_{d}}{M_{d}}\cdot 100\%$$

where *M_s_*—the weight of swollen hydrogel at time *t*, (g); *M_d_*—the weight of dry hydrogel (g).

The equilibrium swelling degree (*Q_eq_*, %) was determined for the never-dried hydrogels (initial hydrogels immediately after synthesis) using Equation (2):

(2) $$Q_{eq}=\frac{M_{\infty }-M_{d}}{M_{d}}\cdot 100\%$$

where *M_∞_*—the weight of swollen hydrogel at equilibrium (g); *M_d_*—the weight of dry hydrogel (g).

In order to determine the kinetic parameters of the water diffusion process, Equation (3) was used to describe the Fickian or non-Fickian behavior of swelling-controlled release systems:

(3) $$\frac{W_{t}}{W_{eq}}=k_{sw}\cdot t^{n_{sw}}$$

where *W_t_*—the amount of water absorbed by the hydrogel at time t, (g); *W_eq_*—the amount of water absorbed by the hydrogel at equilibrium (g); *k_sw_*—the swelling rate constant, characteristic of the 3D network; *n_sw_*—the swelling diffusional exponent, which is dependent on the geometry of the device and indicates the transport mechanism. The constants *n_sw_* and *k_sw_* were calculated from the slopes and intercepts of the plots of ln(*W_t_/W_eq_*) vs. ln(t). Equation (3) was applied in the early swelling stages (swelling degree less than 60%), where linearity was observed. 

#### 2.2.2. Scanning Electron Microscopy (SEM) 

Scanning electron microscopy (SEM) analyses were performed on a Verios G4 UC Scanning Electron Microscope (Thermo Scientific, SEM, FEI Company, Brno, Czech Republic). Cross-sections of the CLE hydrogels were coated with a platinum layer (6 nm) prior to examination, using a Leica EM ACE200 Sputter coater (Leica Microsystem, Vienna, Austria). SEM analyses were conducted using a secondary electron detector (Everhart–Thornley detector, ETD) with 10 kV accelerating voltage and a beam current of 0.8 nA. The average pore size ± standard deviation (SD) was determined for each sample, by measuring 100 randomly chosen pores from the SEM micrographs exported into an image analysis software (ImageJ software, v1.53k).

#### 2.2.3. ATR-FTIR Spectroscopy (ATR-FTIR)

ATR-FTIR investigations were carried out on silicon single-crystal parallelepiped internal reflection elements (IRE; 55 mm × 5 mm × 2 mm, 45° incident angle), using a Bruker Vertex 70 spectrometer (Bruker Optics, Ettlingen, Germany). All the ATR-FTIR spectra were the results of 256 scans at a resolution of 4 cm^−1^, in the frequency range of 4000–400 cm^−1^. 

#### 2.2.4. Rheological Characterization

The rheological properties of the cellulose-based hydrogels were investigated on a Physica MCR-301 Rheometer (Anton Paar, Graz, Austria) at 25 °C, using parallel plate geometry with 25 mm diameter and a 3 mm gap between the plates. Each hydrated hydrogel was placed on the lower plate, the excess sample being removed after the upper plate descended to the desired gap height. A Peltier device was used for temperature control and the prevention of water evaporation. Amplitude sweep tests were performed in the strain range of 0.001–100% at 10 rad/s to determine the linear viscoelasticity region, LVR (stable values for G′ and G″). Frequency sweep measurements were carried out in the frequency range from 0.05 to 500 s^−1^, with a constant strain of 1% (determined from LVR) to evaluate the viscoelastic properties of the cellulose-based hydrogels, such as storage (G′) and loss (G″) moduli, and complex viscosity (η*) as a function of angular frequency. 

#### 2.2.5. Mechanical Properties

The mechanical tests were performed using a Brookfield Texture PRO CT3(R) Analyser (Brookfield Engineering Laboratories Inc., Middleboro, MA, USA).

The compressive test was performed with CLE hydrogels of rectangular shape (20 mm length, 11 mm width, and 9 mm high) at room temperature. The samples were saturated with PBS of pH 5.5 for two hours, then placed between two parallel plates and tested under uniaxial compression till 70% deformation, using a 4500 g load cell and 0.2 mm/s test speed. 

The compressive strength was measured at the 60% strain level, while the elastic modulus (*E*) was calculated from the linear part of the stress–strain curves, between 3 and 10% compression, using Equation (4):

(4) $$E=\frac{\sigma }{\varepsilon }=\frac{\frac{F}{A}}{\frac{\Delta l}{l_{0}}}$$

where *σ*—compressive stress; *ε*—strain; *F*—force (N); *A*—cross-sectional area of the hydrogel (m^2^); Δ*l*—change in length; *l*_0_—original length.

#### 2.2.6. Mucoadhesion Capacity

In order to investigate the mucoadhesion ability of the obtained hydrogels, chicken skin mucosa was used as the model surface. This device (TA-MA kit) consists of a membrane holder, where the mucosal tissue was fixed between two plates. The hydrogel discs (10 mm diameter and 3 mm height) were attached with double-sided adhesive to the lower end of a cylindrical probe (TA5, 10 mm diameter). The skin and the sample were kept in contact for 60 s with 1N force applied during this time. After 60 s, the sample was moved upward (0.5 mm/s) until complete separation between the surfaces occurred, and the mucoadhesive force (Fmax, N) was measured using TexturePro CT Software (TA-CT-PRO-AY). The mechanical measurements were performed in triplicate, and the average of three determinations was used to estimate the elastic modulus and the adhesiveness.

#### 2.2.7. Hydrolytic Degradation

The hydrolytic degradation of the CLE hydrogels was studied in phosphate-buffered saline (PBS, pH 7.4) solution, the most common buffer used in studies for biomedical applications, due to its similarity to bodily fluids. Samples of about 100 mg were immersed in 80 mL PBS and placed in a thermostatic water bath at 37 ± 0.2 °C. The hydrolytic degradation in PBS was monitored for 14 days, when the samples were weighed after removing the excess liquid from their surface. The CLE hydrogels were left to swell under these conditions for 24 h, and then weighed—this was considered the initial swollen weight (*SW*_0_), and the recorded weight over time was noted as *SW*. The weight change of the hydrogel over time (*WC*) was determined using Equation (5):

(5) $$WC=\frac{SW-{SW}_{0}}{{SW}_{0}}$$

where *WC*—weight change of the hydrogel over time (%); *SW*_0_—the initial swollen weight of the hydrogel (g); *SW*—the swollen weight at a specific time (g). 

It should be mentioned that, in this study, only the hydrogels CLE 1 to CLE 6 were chosen, because the hydrogel CLE 7 started to disintegrate after 24 h of swelling. In addition, the pH of the solutions was measured immediately after weighing the samples. 

#### 2.2.8. Incorporation of PrHy Release

In total, 0.1 g dried hydrogel was immersed in 50 mL solution of 4 g/L PrHy and left to swell at room temperature for 72 h, while the PrHy penetrated the 3D network. Then, the PrHy-loaded hydrogels (P-CLE) were dried by lyophilization. In order to establish the amount of PrHy incorporated into the hydrogels, the remaining solutions were analyzed by UV-VIS spectroscopy (Shimadzu UV-1900i, Kyoto, Japan), measuring the absorbance at 290 nm. The calibration curve, y = 71.337x, with a correlation coefficient of R^2^ = 0.999, was established using different PrHy solutions with concentrations between 10^−4^–10^−2^ g/mL. The unknown concentrations of the solutions of PrHy remaining after hydrogel loading were determined using the equation obtained from the calibration curve. 

The incorporation degree (Id) of PrHy into hydrogel matrices was calculated using Equation (6):

(6) $$I_{d}=\frac{c_{0}\cdot V_{0}-c\cdot V}{M}\cdot 100\%$$

where *c*_0_—the initial concentration of the PrHy solution (g/mL); *V*_0_—the volume of initial PrHy solution (mL); *c*—the concentration of PrHy solution after hydrogels loading, determined from the calibration curve (g/mL); *V*—the volume of the PrHy solution remaining after incorporation (mL); *M*—the weight of the dry PrHy-loaded hydrogel (g).

#### 2.2.9. In Vitro Release of PrHy

In vitro release studies were performed in distilled water, at 37 °C, using a standard dissolution procedure. Here, 3 mL samples of release medium were withdrawn periodically, at predetermined time intervals, and the absorbance at 290 nm was measured. In order to maintain the solution’s concentration, the sample was reintroduced into the system after analyzing. The PrHy concentration in the release medium was calculated based on the calibration curve. 

A semi-empirical equation using the Korsmeyer and Peppas model was used to kinetically analyze the data in terms of the PrHy release, such as in Equation (7):

(7) $$\frac{M_{t}}{M_{\infty }}=k_{r}\cdot t^{n_{r}}$$

where *M_t_*—the amount of PrHy released at time *t* (g); *M_∞_*—the amount of PrHy at the equilibrium state (g); *k_r_*—structural/geometric constant for a particular system; *n_r_*—the diffusional exponent, representing the release mechanism. 

#### 2.2.10. Antimicrobial Tests

The antimicrobial activity of the hydrogels was considered against two different strains: *Escherichia coli* (Gram-negative bacteria, ATCC 25922) and *Staphyilococcus aureus* (Gram-positive bacteria, ATCC 25923). This was established by following the standardized methods of bacteriological procedures, according to ISO 16649-2:2001 [19] (*Escherichia coli*) and ISO 6888-3:2003 [20] (*Staphyilococcus aureus*): (i) sterilization of samples in an autoclave for 20 min, at 110 °C and 0.5 bars; (ii) the seeding of the pre-inoculated culture medium, American Type Culture Collection (ATCC) culture bacteria, and incubation for 24 h at 37 °C; (iii) the counting of colonies in 0.1 mL culture by selective culture medium separation; (iv) 0.1 mL of bacterial culture ATCC was inoculated with sterile swabs on the surface of the samples; (v) incubation of samples contaminated with ATCC for 24 h at 25 °C in the dark, in sterilized glass Petri dishes, repeated for another 24 h; (vi) identification of the target germs.

#### 2.2.11. In Vitro Biocompatibility Assessment (MTS Assay)

Human gingival fibroblasts (HGF, CLS Cell Lines Service GmbH, Eppelheim, Germany) were seeded (0.5 × 10^5^ cells/mL) into 96-well plates. The biocompatibility of the samples was assessed using the CellTiter 96® AQueous One Solution Cell Proliferation Assay (Promega, Madison, WI USA), according to manufacturer’s instructions and ISO 10993-5:2009 [21] (extract dilution method). Samples (4 mg/mL) were extracted over 24 h, at 37 °C, in complete cell culture medium: MEM α medium with 10% fetal bovine serum (FBS, both from Gibco, Thermo Fisher Scientific, Waltham, MA USA) and 1% Penicillin–Streptomycin–Amphotericin B mixture (10 K/10 K/25 μg, Lonza, Basel, Switzerland). The cells were incubated with fresh complete medium (Control) or different concentrations of sample extracts (1 mg/mL, 2 mg/mL, 3 mg/mL, 4 mg/mL) for 24 h. MTS absorbance readings were taken at 490 nm on a FLUOstar® Omega microplate reader (BMG LABTECH, Ortenberg, Germany). Experiments were done in triplicate, and the treated cells’ viability was expressed as a percentage of the control cells’ viability (means ± standard deviation).

#### 2.2.12. Statistical Analysis

GraphPad Prism 8 software (GraphPad Software Inc., San Diego, CA, USA) was used for statistical analyses. Data were expressed as mean ± SD and analyzed by independent two-tailed (Student’s) *t*-test, considering *p* < 0.05 as statistically significant.

## 3. Results and Discussion

### 3.1. Preparation of CLE Hydrogels

Lignin is an excellent candidate for chemical modifications, due to its high functionality (the presence of phenolic and aliphatic hydroxyl groups), which gives the possibility of developing new biomaterials [13]. It is expected that the incorporation of LE with high contents of ether bonds would provide higher structural flexibility of the hydrogels, an improved swelling capacity, and also antimicrobial properties [22]. 

Thus, starting from the idea of designing a hydrogel with significantly improved properties and performance, hydrogels containing C and LE were made by the cross-linking reaction in the presence of ECH. ECH is widely used in the cross-linking process of biopolymers, and more importantly, it allows for obtaining polymers free from crosslinker residues, due to the high efficiency of the washing process [23]. Our previous study on the in vitro cytocompatibility of cellulose–dextran hydrogels (CD) provided evidence in favor of ECH and of the safety of materials prepared by chemical cross-linking with ECH. The cell-based experiments showed that human fibroblasts and endothelial cells were successfully cultured on the CD hydrogels with high viability of over 80% for all formulations [12]. Safe and noncytotoxic cellulose-based hydrogels were constructed via chemical cross-linking with ECH for potential applications in biological imaging, when the viability of L02 cells was demonstrated to exceed 90% for all hydrogels [24].

Related to LE preparation, it has been noted that, under the conditions of an alkaline environment (NaOH), the epoxy groups in ECH open, and the cross-linking reaction with the OH groups from lignin takes place. Moreover, after the removal of chlorine, new epoxy groups can be formed, which further react with other OH groups within lignin, achieving the cross-linking of the lignin (LE) [25]. The obtained LEs have an epoxy equivalent of 0.2%, a value confirmed by other authors as well [26,27].

Generally, the cross-linking process with ECH that takes place in various alkaline media is controlled by the synergy between the chemical and physical cross-linking processes of the polymer chains, and more precisely by (i) the chemical cross-linking processes, through the etherification reaction between the OH groups of ECH and those of the cellulose chains, as well as by (ii) the physical cross-linking process between the OH groups of the polymeric chains, with the reconstruction of hydrogen bonds [28]. 

In our particular case of CLE hydrogels, the OH groups of cellulose and those of LE are covalently linked to the reactive groups of ECH, and covalent bonds are established either between two functional groups belonging to the same macromolecular chain, or with the groups of a neighboring polymeric chain. 

**Figure 1:**
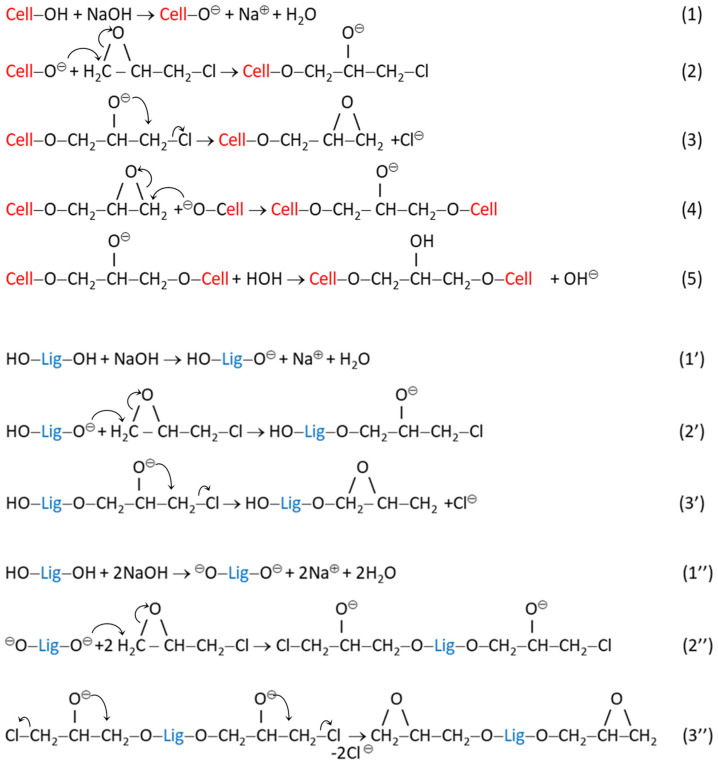

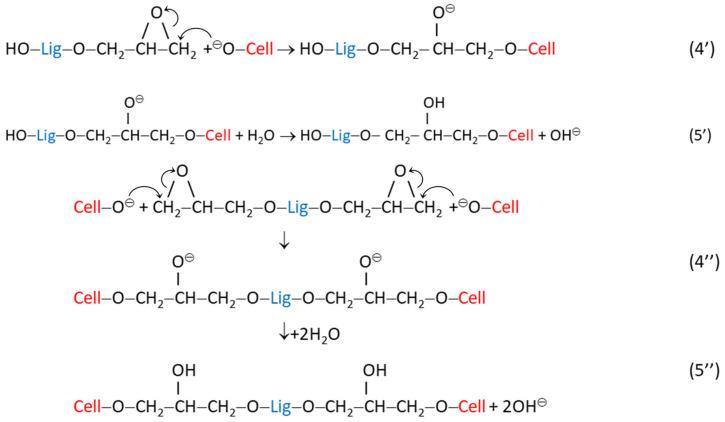
The proposed mechanism of the cross-linking reaction of cellulose with LE, in the presence of ECH: (1)–(5)—possible cross-linking reactions between cellulose (Cell) and ECH; (1′)–(3′) and (1’’)–(3’’)—chemical reactions between lignin (Lig) and ECH; (4′)–(5′) and (4″)–(5″)—possible cross-linking reactions between and Cell, LE and ECH.

The proposed mechanism for the cross-linking process between cellulose and LE with ECH, in alkaline medium, is presented in Figure 1. 

Chemical reactions (1)–(5) illustrate the cross-linking mechanism of cellulose (Cell) with ECH, highlighting the formation of ether bonds between the cellulose chains.

The next reactions, (1′)–(3′) and (1″)–(3″), prove the chemical modification of lignin macromolecules (Lig) by introducing multiple epoxy groups, but also by preserving some unreacted OH groups.

The last group of chemical reactions, (4′)–(5′) and (4″)–(5″), exemplify the possible interactions between the active species based on Cell and Lig in the reaction medium.

In addition, some secondary reactions may occur, in which the groups in ECH could interact only with an OH group from cellulose or lignin, and there is also the possibility of hydrolyzing some ECH. It is worth mentioning that the gravimetric ratio between C and LE is an important factor in the formation of the 3D network of CLE hydrogels, with a strong impact on the characteristics of the hydrogels, especially on their swelling capacity. 

### 3.2. Swelling Behavior of CLE Hydrogels

**Figure 2:**
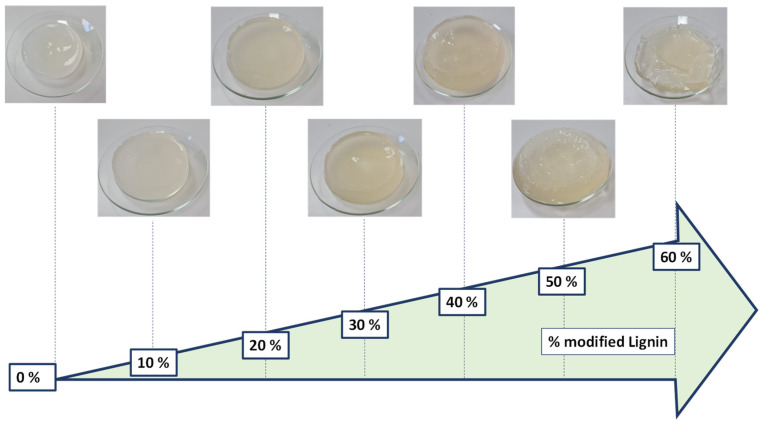
Changes in the appearance of CLE hydrogels with increasing LE content in the matrix.

The color of CLE hydrogels depends on the LE content within the 3D network (Figure 2). Importantly, it has been remarked that, in comparison with the cellulose–lignin (CL) hydrogels, which have a color from light brown to dark brown [17], CLE hydrogels are semitransparent and turn from white to slightly yellowish, with an increase in the LE content in the matrix.

Moreover, an obvious increase in the volume of CLE hydrogels is observed, along with the increase in the content of LE in the system, accompanied by a gradual increase in samples’ *Q_max_*. 

**Figure 3:**
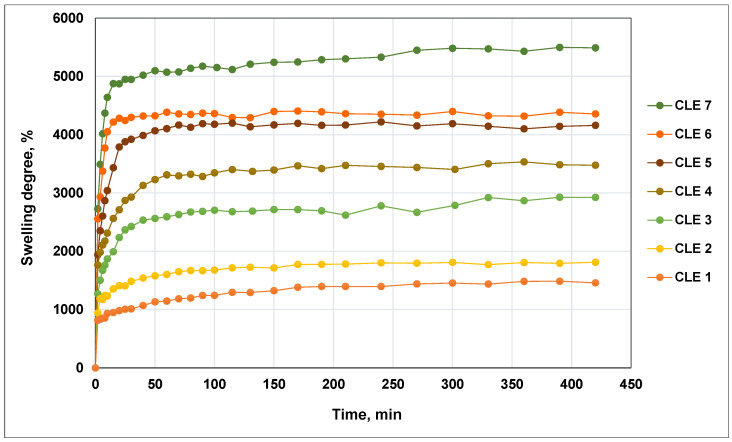
Swelling kinetics of CLE hydrogels as a function of time and composition.

The evolution in time of *Q_max_* as a function of the composition of CLE hydrogels is presented in Figure 3.

It is observed that the swelling properties of CLE hydrogels are influenced by the presence of LE within the hydrogels; thus, a higher content of LE determines a higher swelling capacity. This behavior can be explained by the presence of a large number of polar groups (aromatic and aliphatic hydroxyls, carbonyls) in LE, which are partially involved in C–O–C bonds, but with most hydroxyl groups remaining free and conferring hydrophilicity to the matrix. Also, a contribution to the increase in *Q_max_* is made by the voluminous structure of the LE, which allows for the formation of a more relaxed 3D network and, implicitly, a higher swelling capacity.

The composition of CLE hydrogels, the gel fraction yield and the *Q_eq_* of the hydrogels are presented in Table 1. 

The *Q_eq_* determined for never-dried hydrogels varies from 1920% for CLE 1 up to 10.265% for CLE 7. In addition, the *Q_max_* is improved from 1460% for CLE 1 up to 5490% for CLE 7. It should be noted that CLE hydrogels exhibited a significantly higher swelling capacity than the CL hydrogels reported earlier in the literature [17], suggesting that the use of LE in the preparation of CLE hydrogels greatly improves the capacity for water absorption. Furthermore, if LE is used in the preparation of hydrogels together with poly(vinyl alcohol) (PVA), the same tendency of increasing an *Q_max_* with increasing LE content within the hydrogel is observed, but the *Q_max_* values recorded for LE hydrogels–PVA are significantly lower than those obtained in this study [27].

The kinetic parameters of the swelling of CLE hydrogels are presented in Table 2, where the influence of the hydrogel’s composition on the kinetics of the water uptake process can be observed.

The values of the diffusional exponent (*n_sw_*) are lower than 0.45, indicating that the water diffusion rate is lower than the polymer chains relaxation rate, which shows that the water transport mechanism is defined by the less Fickian diffusion. With the increase in the LE content in the hydrogel formulations, an increase in the *n_sw_* value is observed, showing a tendency towards the Fickian diffusion process, as characterized by *n_sw_* = 0.45.

The swelling rate constant (*k_sw_*), which characterizes the diffusion rate in the hydrogels network, varies with the composition; more precisely, it increases with the increase in the LE content in hydrogel matrices.

For all hydrogels, the correlation coefficients R^2^ are higher than 0.99, indicating a good fitting between experimental data and the chosen model.

### 3.3. Scanning Electron Microscopy (SEM)

**Figure 4:**
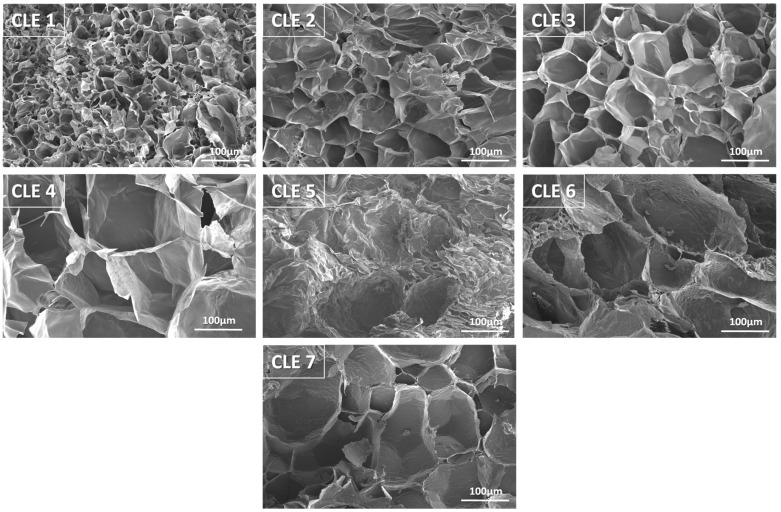
SEM micrographs of CLE hydrogels (magnification 250×).

The morphological details of all CLE hydrogels were assessed using SEM micrographs, as presented in Figure 4. The cross-section SEM images reveal that all prepared hydrogels formed a macroporous heterogenic structure, with interconnected pores of various sizes. 

It can be observed that a higher content of LE in the CLE hydrogels determines evident changes in pores size and pore distribution within the 3D matrices, which are critical factors in the control of swelling capacity and drug release behavior. It is easy to observe that an increase in the LE content results in hydrogels with lower pore density and higher average pore size, namely, hydrogels CLE 4–CLE 7 having larger, more irregular pores, as compared to hydrogels CLE 1–CLE 3. Thus, the presence of LE in the hydrogels determines the formation of a more heterogeneous, loosely packed porous architecture, compared with CLE 1, where the porous architecture seems to be denser. Moreover, by increasing the LE content, it is observed that the walls between the pores become thinner, leading to the formation of a more relaxed porous network, capable of retaining a larger amount of water, observations that are confirmed by the data regarding the swelling degree of the CLE hydrogels. 

The average pore sizes increased gradually from CL1 to CL7 and range from 38.2 ± 15.2 μm (CLE 1) to 63.3 ± 25.2 μm (CLE 2), 71.0 ± 28.1 μm (CLE 3), 129.3 ± 52.5 μm (CLE 4), 131.4 ± 41.9 μm (CLE 5), 137.4 ± 45.4 μm (CLE 6) and 148.0 ± 43.0 μm (CLE 7), respectively. The difference between the diameters of pores in CLE 1 (without LE) and CLE 7 (with the greatest amount of LE) is noticeable.

**Figure 5:**
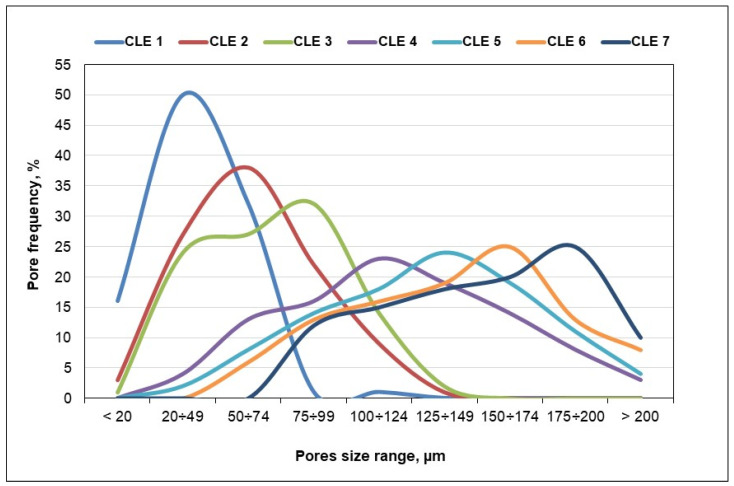
Pore size distribution of CLE hydrogels.

Figure 5 shows the pore size distribution for CLE hydrogels. It can be observed that, as the LE content increased in the CLE hydrogels, a wider distribution of the pore size was registered (CLE 4–CLE 7), compared to those containing a smaller amount of LE (CLE 1 and CLE 2). For example, if, for CLE 1, the pore size frequency is concentrated in a small size range, between 20 and 75 µm, in the case of sample CLE 7, this range widens, starting from 75 µm and continuing up to approximately 275 µm, with a maximum size located in the range of 175–200 µm. In fact, with the increase in the LE content in the hydrogel, the size range moves to the right with the greater pore frequency, to larger sizes, with approximately 25 µm for every 5% addition of LE in the formula. 

In this way, by changing the LE content in the hydrogel formula, the size and frequency of the pores in the matrix can be easily controlled, and implicitly, all the properties of the hydrogel that are directly influenced by these parameters.

### 3.4. ATR-FTIR Spectroscopy

The structural changes of the CLE hydrogels following the incorporation of different amounts of epoxidized lignin in the 3D network were investigated by ATR-FTIR spectroscopy. 

**Figure 6:**
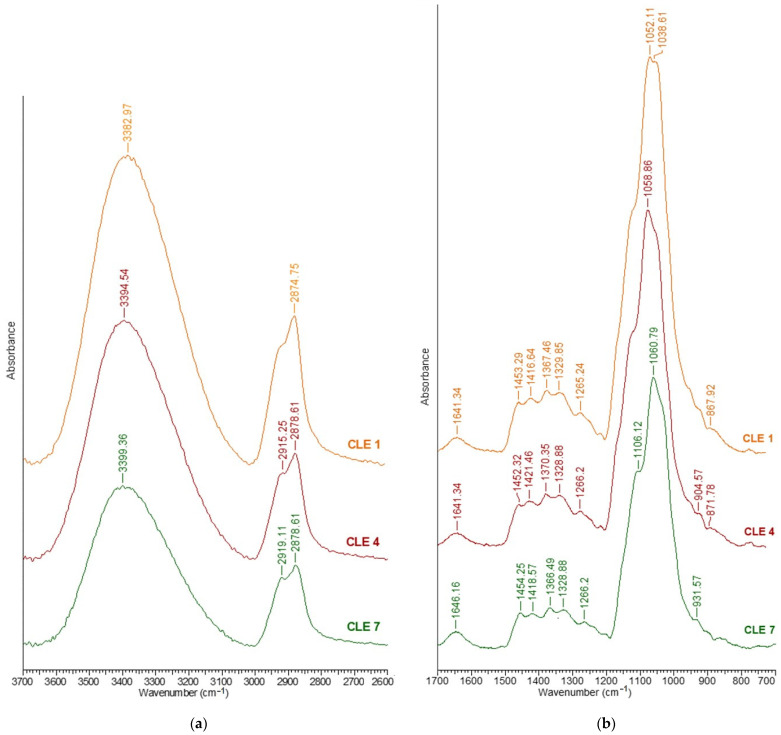
ATR-FTIR spectra of CLE hydrogels in (**a**) 3700–2500 cm^−1^ region and (**b**) 1700–700 cm^−1^ region.

Due to their complexity, the spectra are presented into two regions, such as: (i) 3700–2600 cm^−1^, the region of the OH and CH stretching vibrations (Figure 6a), and (ii) 1700–700 cm^−1^, the “fingerprint” region, assigned to the stretching vibrations of different groups (Figure 6b). From all the spectra, only three have been selected to be presented, namely, the spectra of CLE 1 (C:LE = 100:0), CLE 4 (C:LE = 70:30) and CLE 7 (C:LE = 40:60).

A broad band assigned to OH stretching at 3700–3000 cm^−1^ is observed for all the spectra (Figure 6a), and also, a shift of the maximum absorbance to higher wavenumbers, from 3382 cm^−1^ (CLE 1) to 3399 cm^−1^ (CLE 7), was recorded. This band is more prominent for the CLE 1 sample than for the other samples, which is due to the presence of a large number of hydroxyl groups being involved in an increased number of hydrogen bonds, considered to be the cause of this broad OH band. By increasing the amount of LE in the matrix, the absorbance of OH stretching decreases progressively, proving the lower involvement of -OH groups in new hydrogen bonds, or perhaps even the scission of the already formed intra- and inter-molecular hydrogen bonds.

In the region of 3000–2800 cm^−1^, two strong bands can be observed for CLE 1 at 2912 cm^−1^ and 2874 cm^−1^, respectively, which can be attributed to the stretching of symmetric and asymmetric methyl and methylene groups. The appearance of the band at 2892 cm^−1^, attributed to CH stretching, demonstrates the modification of the cellulose crystal system following the process of obtaining the hydrogel; more precisely, the transition from cellulose I to cellulose II in the presence of the NaOH aqueous solution [29]. The two bands that appeared in the samples with different LE contents (CLE 2 to CLE 7), namely, 2919 cm^−1^ (symmetric) and 2879 cm^−1^ (asymmetric) for CLE 7, can also be attributed to the CH stretching vibration in the aromatic methoxyl group and the methyl and methylene groups [30]. 

Moreover, it was observed that, by increasing the content of LE within the hydrogels, obvious decreases in the band at 2919 cm^−1^, characteristic of asymmetric CH stretching vibrations, and in the band at 2879 cm^−1^ were recorded.

In the 1700–700 cm^−1^ region, a complex region of the ATR-FTIR spectrum of the hydrogels, several characteristic bands can be identified, among which we might mention: (i) the band at 1635 cm^−1^, which is attributed to adsorbed water; (ii) the absorption band at 1419 cm^−1^ (characteristic of cellulose II [29], attributed to the symmetric CH_2_ bending vibration—this band is also known as the “crystallinity band” [17] and a decrease in its intensity, as can be seen in Figure 6b, indicates a reduction in the crystallinity degree of the samples; (iii) the band at 1453 cm^−1^ attributed to the CH_2_ and CH groups of cellulose; (iv) the band at 1367 cm^−1^ attributed to the symmetric CH_2_ bending vibration; (v) the band at 1266 cm^−1^ attributed to the CH bending vibration; (vi) the band at 1162 cm^−1^ attributed to C–O–C groups at the *β*-glycosidic linkage; (vii) the band at 1053 cm^−1^ attributed to the CO bending vibration at C3; (viii) the band at 1038 cm^−1^ attributed to the CO bending vibration at C-6 and (ix) the band at 867 cm^−1^ attributed to C–O–C stretching at the β-(1→4)-glycosidic linkages.

In the "fingerprint" region, the bands characteristic of cellulose overlap with those characteristic of LE, and therefore, it is difficult to distinguish between them. 

However, it should be mentioned that the bands at 1454 cm^−1^ and 1508 cm^−1^, characteristic of CLE hydrogels, are attributed to asymmetric CH deformations in the methyl, methylene and methoxyl groups, and to the aromatic vibrations of the skeleton coupled with CH in plane strains, respectively [30]. Also, the bands at 1329 cm^−1^ can be due to the C=O stretch of the syringyl ring, at 1266 cm^−1^ to the C=O stretching of the syringyl ring and at 1217 cm^−1^ to the C=O stretching of the guaiacyl ring. The characteristic bands of LE, where the presence of epoxy groups is highlighted, appear at 1230 cm^−1^, 910 cm^−1^ and 830 cm^−1^ [26].

The broad band at 3400 cm^−1^ of the samples with an increased content of LE is also attributed to the stretching vibrations of phenolic and aliphatic hydroxyl groups involved in hydrogen bonds, while the bands at 2919 cm^−1^ and 2879 cm^−1^ are attributed to the CH stretching in methyl of aromatic and methylene groups of side chains and aromatic methoxyl groups.

As can be seen in Figure 6a, there is a decrease in the band at 3400 cm^−1^, related to OH stretching, which is due to the decrease in the number of newly formed hydrogen bonds, as a result of the increasing amounts of LE within the matrix. To prove this observation, several structural parameters were inferred from the ATR-FTIR spectra, such as the hydrogen bond intensity (HBI), hydrogen bond energy (*E_H_*), the enthalpy of the *H*-bond formation (Δ*H*) and the asymmetric index (a/b), data that are presented in Table 3.

To establish the hydrogen bond intensity (HBI), the ratio between the absorption bands at 3400 cm^−1^ and 1320 cm^−1^ is used [31]. This parameter is an indicator of the mobility of the chains, and more precisely, of the structure’s ordering degree (crystallinity), and also of the amount of bound water [29]. From the data presented in Table 3, it can be inferred that the HBI value for CLE 1 (5.28) is the highest, which demonstrates the presence of a large number of intra- and intermolecular hydrogen bonds, which determine a more compact structure, with better organization and greater crystallinity of the sample. This intensity gradually decreases from 5.28 (CLE 1) to 4.36 (CLE 4), until 3.95 for CLE 7, indicating that the introduction of LE leads to a distortion of the sample’s organization, and implicitly to a decrease in the number of hydrogen bonds and the crystallinity of the sample. 

To confirm this assumption, the energy of the *H*-bonds (*E_H_*, kJ) has been determined. This parameter is calculated using Equation (8) [17]:

(8) $$E_{H}=\frac{1}{k}\cdot \frac{\nu_{0}-\nu }{\nu_{0}}$$

where *ν*_0_—the standard frequency corresponding to free -OH groups (cm^−1^); *ν*—the frequency of the bonded -OH groups (cm^−1^); *k* = 1.68 × 10^−2^ kcal^−1^. 

The values obtained for *E_H_* follow the same trend as those of HBI, which confirms the reduction in the number of hydrogen bonds in the supramolecular structure of the hydrogels—a gradual decrease from CLE 1 to CLE 7 with the increase in the addition of LE. 

The LE addition in the 3D networks causes a decrease in the a/b index values, a fact that confirms the information obtained when determining the other parameters (HBI and *E_H_*), namely, that the uniformity of the sample decreases from CLE 1 to CLE 7.

The enthalpy of *H*-bond formation (Δ*H*, J/g) was calculated with Equation (9) [17]:

(9) $$-∆H=0.016\cdot ∆\nu_{OH}+0.63$$

where Δ*ν_OH_*—the difference between the frequency corresponding to free -OH groups (cm^−1^) and the frequency of the bonded -OH groups (cm^−1^). 

The obtained Δ*H* values (Table 3) indicate that the number of hydrogen bonds established between the -OH groups in CLE 1 is greater than those established between the -OH groups in CLE 7—data that confirm the previously presented assumptions.

For a supplementary investigation of the hydrogels’ crystalline structure, we went further and determined the two relative absorbance ratios usually applied to study crystallinity changes, which are total crystalline index (TCI) and lateral order index (LOI) (Table 3). The total crystalline index (TCI) was calculated from the ratio between the absorption bands at 1375 cm^−1^ (CH_2_ bending vibration) and 2892 cm^−1^ (CH stretching vibration). This ratio is proportional to the crystallinity of the samples, and its decrease indicates a decrease in the crystallinity degree of the samples. In our case, the highest crystallinity ratio was recorded for CLE 1 (0.55), and then the crystallinity ratio of the hydrogels consistently decreased to 0.53 (CLE 4) and 0.48 (CLE 7).

Another important ratio is the lateral order index (LOI), which was established as the ratio between the absorption bands at 1420 cm^−1^ (symmetric CH_2_ bending vibration) and 893 cm^−1^ (β-1,4-glycosidic bonds in cellulose). For the prepared cellulose-based hydrogels, which presented the crystalline structure of cellulose II, the ratio with the band at 893 cm^−1^ was used, because this is the band used for the samples predominantly composed of cellulose II [32,33]. This index is correlated to the overall degree of order in the sample, and, for the special case of cellulose II, a lower value reflects a more ordered structure [32,34]. It was observed that the LOI values increase with the decrease in the TCI, which is proportional to the crystallinity degree of the hydrogels. More exactly, CLE 1 has the highest TCI index and the lowest LOI index in comparison with CLE 7, which has the highest LOI index and the lowest TCI index.

The formation of etheric bonds in the hydrogels, following the cross-linking reaction between C and LE, was highlighted by the aryl ether band (C–O–C stretching) at 1266 cm^−1^ and the alkyl ether bands at 1106 cm^−1^ (C–O–C stretching) and 1060 cm^−1^ (C–O stretch from alkyl substituted ether). In order to highlight the structural changes that occur when the LE content increases in the CLE hydrogels, the ratio between the ether bands mentioned above and a band considered an internal standard for cellulose II, the band at 2892 cm^−1^, was determined (Table 4). The formation of etheric cross-linking bonds was highlighted for all CLE hydrogels. It is worth mentioning that the number of these bands decreases with the increase in the LE content. A possible explanation for this behavior lies in the fact that, following the chemical modification of lignin, some free -OH groups are blocked with epoxy groups, which prevents the formation of new cross-linking bonds. 

The characteristic band of lignin, at 1508 cm^−1^, attributed to aromatic skeletal vibrations, is independent of the ones related to cellulose. Thus, in order to establish the LE content within the CLE hydrogels, a ratio between this band and the one at 2892 cm^−1^ was used [35]. Notably, the treatment with NaOH solution decreases the intensities of the band at 1508 cm^−1^, and this is why it is significantly diminished in the ATR-FTIR spectrum of the CLE hydrogels. However, the values obtained for the A_1508_/A_2892_ ratio confirm the presence of LE in the structure of the hydrogels and, in addition, it is observed that the ratio increases as the LE content increases in the CLE samples.

### 3.5. Rheological Investigations

**Figure 7:**
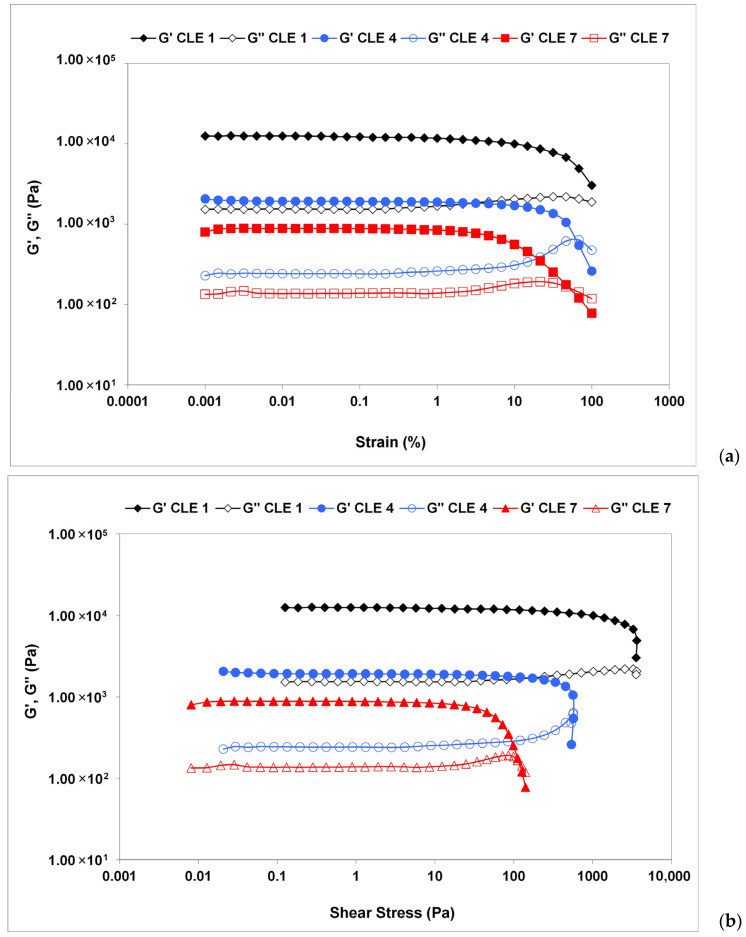
Amplitude sweep test results for CLE 1, CLE 4 and CLE 7 hydrogels: (**a**) G′ and G′′ dependence on the oscillatory strain; (**b**) G′ and G′′ dependence on the shear stress.

The rheological properties of the CLE hydrogels were evaluated by amplitude and frequency sweep tests. The values of the storage modulus (G′) were greater than those of the loss modulus (G″), revealing the gel-like behavior of the CLE hydrogels, which can be thus considered viscoelastic solid materials (Figure 7). An enhanced stiffness “at rest” (G′LVR) has been observed for CLE 1, or the samples containing less LE, as the mean values of G′ within the LVR were found to be of 13,500 Pa for CLE 1, 2560 Pa for CLE 4 and 750 Pa for CLE 7 [36]. 

The curves plotted in Figure 7 show that the dynamic moduli G’ and G" depend on the strain amplitude at values exceeding LVE, the decrease in G’ and G" indicating material breakdown at large deformations. While G’ decreases sharply, G″ increases before reaching a maximum peak, followed by a sudden drop. The rise in G″ is associated with the occurrence of micro-cracks, while the overall structural integrity is maintained as G′ remains constant and dominant over G″ (Figure 7a) [37,38].

The value of shear stress at the limit of the LVE region is considered the yield point (or linearity limit), while the flow point is represented as the value of shear stress at the crossover point of G′ and G″ (where G′ = G″). Between the yield stress and the flow point, with G′ still having higher values than G″, the initial structural strength of the hydrogel is modified, but it still exhibits the properties of a solid material. Above the flow point, G″ becomes greater than G′, and any further increase in shear stress leads to material flow, with the sample acquiring liquid-like properties [39]. The hydrogel CLE 1 did not show a flow point, maintaining its viscoelastic solid properties. The yield stress, stress and strain at the flow point were extracted from amplitude sweep measurements, the resulting values being presented in Table 5. The studied CLE hydrogels showed a flow point over 115 Pa (CLE 7). Increasing the LE concentration led to a decrease in yield stress and flow point values, respectively, with the samples becoming softer, changing their solid network structure.

**Figure 8:**
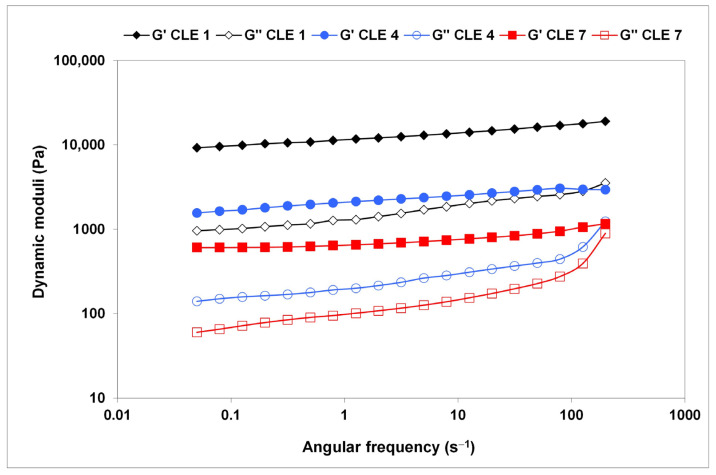
Variation of storage modulus G′ (solid symbols) and loss modulus G″ (open symbols) as a function of oscillatory frequency, ω, at 25 °C.

The results of the frequency sweep tests carried out in the linear viscoelastic region also suggest the gel-like behavior of the CLE hydrogels, as the G′ values exceed those of G″ (Figure 8). A very small variation in G′ was observed in the whole range of investigated oscillation frequencies, in the absence of crossover frequency, demonstrating that the CLE hydrogels display a structured 3D network. 

However, a higher dependency has been registered for G″, with the lowest values of dynamic moduli being recorded for CLE 7, which proved to be the softest material, although maintaining its stable structure. Similar behavior, typical of soft glassy materials, was reported for oxidized pullulan–dopamine cryogels [40].

A stronger and stiffer structure of CLE 1 is revealed by the highest values of G′ and G″ at the tested oscillation frequencies, with about one order of magnitude increase compared to CLE 7. 

### 3.6. Mechanical Properties of CLE Hydrogels

The mechanical properties of CLE hydrogels were determined by a compression test, and the response of CLE hydrogels to compressive stress is an important indicator for their medical applications. 

**Figure 9:**
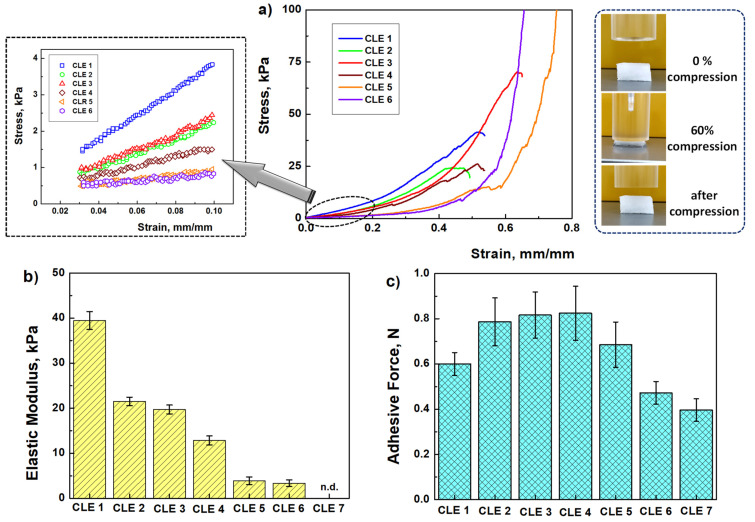
Mechanical properties of CLE hydrogels: (**a**) stress–strain profiles (in detail, linear dependence of stress–strain curves from which the elastic modulus of hydrogel samples was calculated), (**b**) the elastic modulus values as a function of hydrogel’s composition, and (**c**) mucoadhesion force of CLE hydrogels.

Figure 9a presents the compressive stress–strain curves of the CLE measured by a dynamic mechanical analyzer (DMA) with 60% strain in compression. All CLE hydrogels have a compressive strength up to 42% of the original height without fracture. The values of the elastic modulus were calculated at between 3 and 10% compression, where the stress–strain curves are linear (detail in Figure 9a), and the values obtained for the elastic modulus are presented in Figure 9b. 

As expected, the maximum value of the elastic modulus of 39.46 kPa was obtained in the case of the CLE 1 hydrogel, when the structure of the hydrogel was based only on covalent bonds between cellulose chains. Increasing the LE content in the hydrogel’s structure, from 10% (CLE 2) to 30% (CLE 4), caused a decrease in the compressive modulus from 21.52 kPa to 12.86 kPa, respectively, due to the fact that the presence of LE hinders the formation of covalent bonds between cellulose chains. As a result, the elastic modulus of the hydrogels also decreases. In addition, by increasing the LE content to 40–50% (CLE 5 and CLE 6), the elastic modulus value significantly decreases to 3.34 kPa. A higher LE content (CLE 7) resulted in a very soft hydrogel, which did not allow the compression behavior to be recorded on the apparatus.

It is known that values of the modulus of elasticity between 3 and 20 kPa are ideal for oral wound dressings, which need to be soft and flexible [41,42]. This observation suggests CLE hydrogels with a higher LE content be applied as wound healing dressings.

### 3.7. Mucoadhesive Properties

For topical administration to the oral mucosa, polymer hydrogels must adhere to the mucosa, and in this respect adequate mucoadherence is essential. Figure 9c shows the values obtained for the adhesive force of CLE hydrogels, and it can be seen that all the samples fall within the necessary range allowed for the adhesion process (0.3–1.3 N) of oral dressings [43].

As a conclusion, the results obtained for the mechanical tests suggest the CLE hydrogels that have in their composition an LE amount of 10–30%, such as the CLE 2, CLE 3 and CLE 4 hydrogels, as ideal materials for applications as oral wound dressings.

### 3.8. Hydrolytic Degradation of CLE Hydrogels

**Figure 10:**
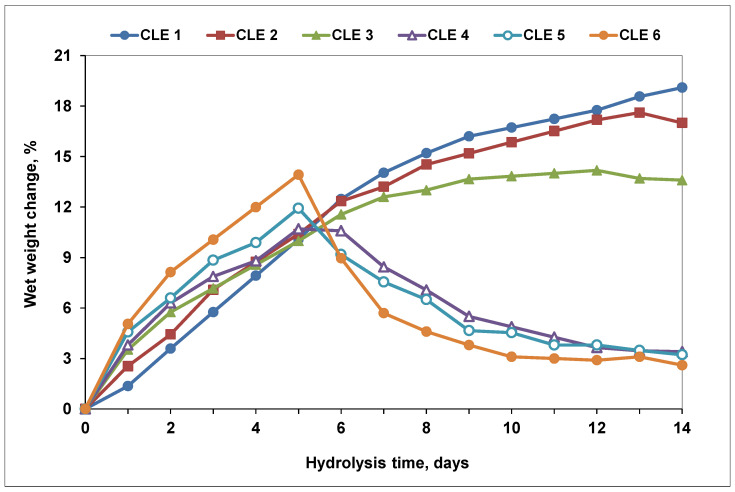
Wet weight change of CLE hydrogels in PBS solution (pH 7.4, 37 °C), over 14 days.

Figure 10 shows the weight change recorded for wet hydrogels, as a function of immersion time. It is observed that, in the first 5–6 days, all CLE hydrogels go through a swelling phase, and after that CLE 5 and CLE 6 begin to degrade, a phenomenon illustrated by the mass decrease. At this point, due to the increase in water content inside the hydrogel, the ether bonds undergo hydrolysis and could be more easily cleaved [44], leading eventually to the detachment of small fragments from the hydrogel matrix. This phenomenon is more accentuated as the LE percentage in the hydrogel increases. Thus, the first hydrogels that start to degrade on the 6th day are those with 40% and 50% LE, respectively—CLE 5 (with 2.8%) and CLE 6 (with approximately 5%). The percentage of mass loss increased with the increasing the immersion time. Thus, the degradation process continues in the following days, reaching on the 14th day a decrease of 7.3% for CLE 4, of 8.7% for CLE 5 and 11.3% for CLE 6, respectively. However, for the other three hydrogels, CLE 1–CLE 3, this hydrolytic degradation does not occur, and in their case only the swelling phase is recorded. This is possible because, in the case of hydrogels with lower LE contents, the swelling process occurs more slowly, and thus the ether bonds are not yet completely hydrolyzed enough to be broken. So, it can be said that the hydrolytic degradation speed of CLE hydrogels is affected by the swelling ratio. 

On the other hand, the pH of the immersion solutions does not vary much during the 14 days; there is only a slight, almost negligible, increasing tendency with the increase in the immersion time.

In conclusion, the hydrolytic degradation study indicates that the CLE hydrogel undergoes small in vitro degradation in the PBS buffer solution over 14 days (i.e., between 7.3 and 11.3%), significantly depending on the LE percentage within the hydrogel, which, in turn, directly influences its swelling behavior. Thus, considering the reduced hydrolytic degradation and their capacity to release drugs in a controlled manner, we can conclude that the proposed hydrogels are suitable for use as oral wound dressings. 

### 3.9. In Vitro Release of PrHy from P-CLE Hydrogels

The degree of incorporation (Id, %) of procaine hydrochloride (PrHy) in CLE hydrogels was studied by UV-VIS spectroscopy. In order to establish the amount of incorporated PrHy in each CLE hydrogel, the UV-VIS spectra of the aqueous solutions of PrHy remaining after the incorporation were recorded at 290 nm. A decrease in the absorption band was observed, which demonstrates an increase in the PrHy content in the P-CLE hydrogels. Moreover, it was found that the amount of incorporated PrHy depends on the composition of the hydrogel and, implicitly, on the *Q_max_* of the CLE hydrogels. Thus, an increase in the LE content in the hydrogel’s composition determines an increase in the Id of PrHy, which can be correlated with the data obtained for the swelling capacity of the CLE hydrogels (Table 6). 

**Figure 11:**
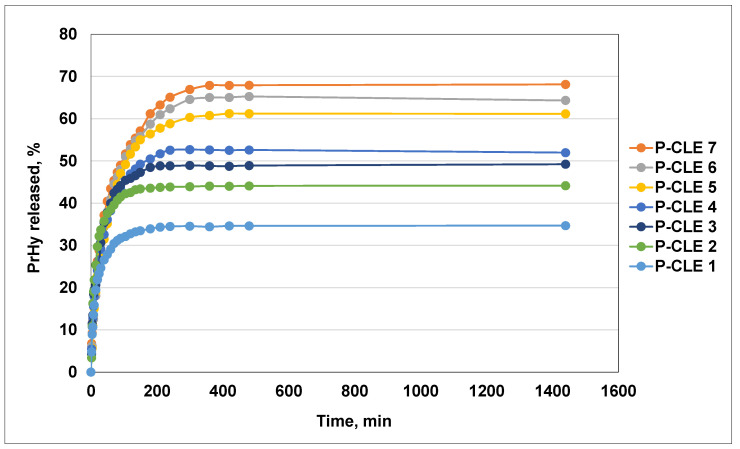
Release profiles of PrHy in distilled water at 37 °C from CLE hydrogels.

The release profiles of PrHy in distilled water at 37 °C from P-CLE hydrogels are presented in Figure 11. As expected, an increase in the swelling degree of the CLE hydrogels leads to a greater uptake of PrHy during the incorporation process and, implicitly, to the release of a greater amount of PrHy. 

It is known that drugs are released from the polymer network only by a diffusion mechanism, and in this sense, the type of porous structure of the hydrogels is particularly important [45]. Depending on the pore size of the 3D network of the CLE hydrogels, which are between 38 μm (CLE 1) and 148 μm (CLE 7), it can be concluded that the CLE hydrogels have a macroporous structure, and that the release mechanism of PrHy is governed by the porosity of the matrix and the diffusion coefficient of the drug. It is observed that the release rate of PrHy is higher for the hydrogel CLE 7, with the highest content of LE, compared to CLE 1, which does not contain LE. Lower hydrogel density and larger pores are the reasons for the faster release. 

The PrHy release profiles indicate for all P-CLE hydrogels a prolonged release of the drug, which increases gradually until about 400 min, where a plateau is reached. The drug is constantly released until 1440 min. There is an obvious influence of the LE content of P-CLE hydrogels on the PrHY release process; more precisely, the increase in the LE content within the matrix determines an increase in the amount of PrHy released. In this regard, it is observed that 68% PrHy is released from the P-CLE 7 hydrogel (with the highest amount of LE), in comparison with 34% PrHy released from the P-CLE 1 hydrogel, whose composition is based only on cellulose. 

To evaluate the possible release mechanism of PrHy from the P-CLE hydrogels and the kinetic parameters of the release process, the experimental release data were investigated by fitting to the Korsmeyer–Peppas model. The kinetic parameters of the release process of PrHy (in distilled water, at 37 °C) from the P-CLE hydrogels are presented in Table 6. 

Thus, the diffusion exponent, *n*, which is an indicator of the release mechanism, and the kinetic constant, *k*, which describes the structural and geometric parameters of the hydrogels, were determined. It is known that, for the hydrogels (cylindrical geometry), the exponent *n* < 0.45 indicates a release mechanism designed as Fickian diffusion, while if 0.45 < *n* < 0.89, the mechanism is defined by non-Fickian diffusion or anomalous transport; when n = 0.89, the release is Case II transport, and for *n* > 0.89, the release mechanism is super Case II transport [46,47].

As can be seen from Table 6, in our case, all P-CLE hydrogels are governed by non-Fickian transport, where both the water diffusion rate and the polymer chain relaxation rate control the overall drug release process, because *n_r_* has values between 0.503 (P-CLE 7) and 0.602 (P-CLE 1). 

It can be concluded that the amount of PrHy released in a given period of time from the CLE hydrogels can be controlled by selecting specific preparation conditions of the hydrogels, such as a certain pore size or cross-linking density, a particular swelling degree and, implicitly, a certain degree of drug incorporation.

### 3.10. Antibacterial Properties of CLE and P-CLE Hydrogels

The oral flora is continuously changing due to the fact that the oral cavity comes into direct contact with the external environment. The species that we can find in the oral cavity, under normal conditions, belong to the genera Streptococcus, Lactobacillus, Lactococcus, Enterococcus, Staphylococcus, Corynebacterium, Veillonella and Bacteroids [48]. Both Gram-positive and Gram-negative bacteria produce bacteriocins, which consist of long peptide chains produced by bacterial ribosomes, used to inhibit or kill another bacterium, in the competition for nutrients within this ecosystem. Gram-negative bacteria have been observed to be more resistant to the bacteriocins produced by Gram-positive bacteria, due to their outer membrane, which acts as an effective barrier [49]. This is the reason why, in the oral cavity, Enterobacteriaceae is the overall predominant family (19.36%), and within this family, the most prevalent species is *Escherichia coli* (53.06%) [50].

Under specific conditions, these oral bacteria can cause numerous systemic infections, including bacterial endocarditis, respiratory pneumonia, osteomyelitis, and even cardiovascular diseases [48]. 

In order to test the antimicrobial activity of the CLE and P-CLE hydrogels, two different strains were considered: *Escherichia coli*, as the model Gram-negative bacteria, and *Staphyilococcus aureus*, as the model Gram-positive bacteria (Table 7). 

The antibacterial activities of the CLE hydrogels were found to be dependent on the amount of LE within the matrix; more exactly, it is higher with the increase in LE content. The same observation was made by El-Nemr and coworkers [51], who explained that the presence of lignin induces the antimicrobial activity of PVA/gelatin–lignin blends due to the content of hydroxyl groups and methoxyl groups that interact with the bacterial cell membrane, causing the disruption and rupture of the cell membrane structure, and finally causing the infiltration of the cell component and the release of the bacterial cell contents. Moreover, the antibacterial effect of lignin modified with epoxy groups was highlighted by Kaur and colleagues, who showed that it is the most effective compared to unmodified or variously modified lignin, against both Gram-negative and Gram-positive bacteria [52].

In addition, CLE hydrogels were found to be more active against *Escherichia coli* than against *Staphylococcus aureus*. The same behavior was stated by Medina et al. [53], who showed that the inhibition effect of lignin on the Gram-positive bacteria was remarkable, compared to that on Gram-negative bacteria. This behavior was explained by the absence of the secondary cell wall of Gram-positive bacteria. 

Furthermore, by testing the P-CLE hydrogels against *Escherichia coli* and *Staphylococcus aureus*, an improvement in the antimicrobial properties of the CLE hydrogels induced by the presence of PrHy in the matrix was observed. When increasing the concentration of PrHy in the hydrogels, from P-CLE 1 to P-CLE 6, the inhibitory effect is more obvious. Also, it was observed that PrHy was more active against *Escherichia coli* than against *Staphylococcus aureus*.

Based on the obtained results, it can be concluded that the hydrogels CLE 4 and CLE 6 are more effective against *Escherichia coli* and *Staphylococcus aureus* than the other hydrogels, which evidences the real potential of these materials to be used as antibacterial wound dressings for administering medical treatments. 

### 3.11. In Vitro Biocompatibility of CLE and P-CLE Hydrogels (MTS Assay)

Human gingival fibroblasts (hGFs) are the main cells of the gingival tissue that play an important role in wound healing, being much more efficient at remodeling connective tissue than dermal fibroblasts. They play an important role in the synthesis of extracellular matrix (ECM) components (collagen, fibronectin, hyaluronan and elastin) and in maintaining tissue integrity [54]. 

To investigate the influence of the amount of LE within CLE hydrogels on their cytocompatibility, hGFs were used. In addition, the cytocompatibility of P-CLE hydrogels was also investigated.

**Figure 12:**
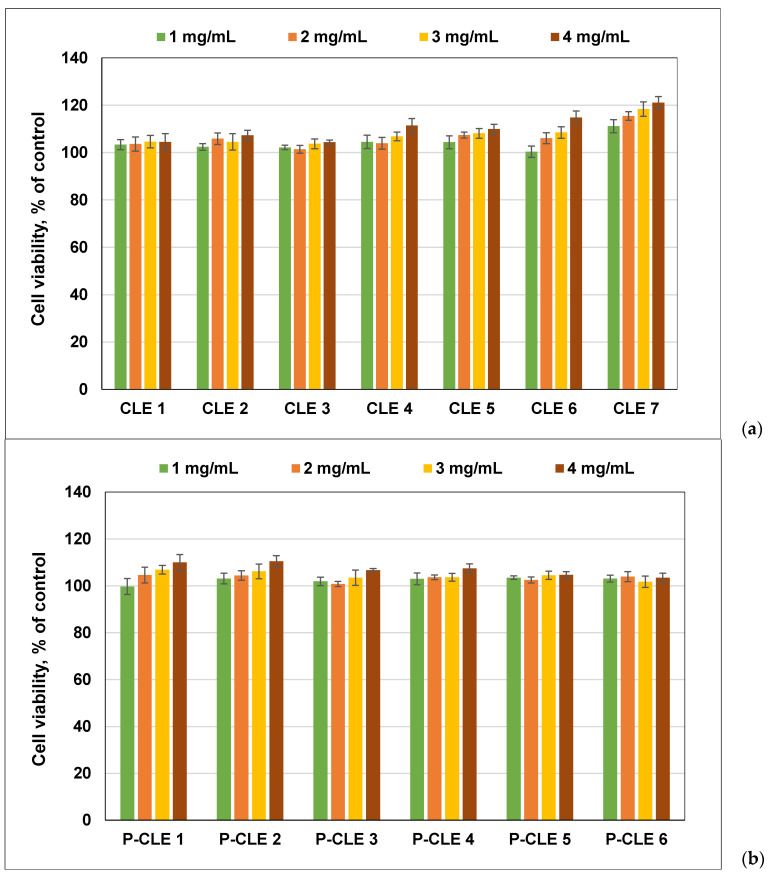
Biocompatibility on human gingival fibroblast cells of (**a**) CLE hydrogels and (**b**) P-CLE hydrogels. Experiments were done in triplicate and treated cell viability was expressed as percentage of control cells’ viability. Graphical data were expressed as means ± standard deviation.

The in vitro biocompatibility of CLE and P-CLE hydrogels was evaluated by the MTS assay, after incubation for 24 h (extract dilution method: extracts at different concentrations of 1 mg/mL, 2 mg/mL, 3 mg/mL and 4 mg/mL), using hGFs (Figure 12).

As already reported, cellulose hydrogels have excellent cytocompatibility for fibroblast cells [12,55]. In our study, the results show that the cellulose hydrogel (CLE 1) has good cell viability, with more than 100% cell viability, regardless of the concentration of the extract used. An increase in the cell viability for the CLE hydrogels is observed through the gradual increase in the percentage of LE in the hydrogel composition, and moreover, this increase in LE also causes the stimulation of fibroblast proliferation up to a maximum value, as recorded for the CLE 7 hydrogel (Figure 12a). Also, increasing the concentration of the extracts up to 4 mg/mL, for each sample, led to an increase in the viability of the fibroblast for each individual sample. 

P-CLE hydrogels were also evaluated regarding their level of safety, by incubating extracts of different concentrations, for 24 h in the presence of fibroblast cells (Figure 12b). It was demonstrated that the presence of PrHy in the P-CLE hydrogels did not significantly change their biocompatibility; thus, cell viabilities of over 99% were obtained. However, a decrease in cell viability was observed for P-CLE hydrogels, compared to CLE hydrogels. In addition, for P-CLE hydrogels, a slight decrease in cell viability was observed, with the increase in the amount of LE in the samples and, implicitly, with the increase in the drug concentration within the 3D networks.

Considering that none of the tested extract dilutions decreased the cell viability to below 99%, according to the international standard ISO 10993-5:2009 [21], it can be concluded that all CLE and P-CLE hydrogels can be regarded as non-cytotoxic materials.

## 4. Conclusions

New multifunctional hydrogels based on cellulose and modified lignin (CLE) have been prepared to be used in advanced wound management.

Through ATR-FTIR spectroscopy, the achievement of the cross-linking reaction between the two natural polymers was confirmed, through the appearance of adsorption bands characteristic of the formation of new ether bonds. It was demonstrated by SEM that the LE contributes to the formation of a more heterogeneous porous architecture, with larger and more irregular pores, compared to the cellulose-based hydrogel (CLE 1). This morphological aspect contributes to the high swelling degree of CLE hydrogels, and also to their superior PrHy encapsulation capacity compared to CLE 1. It was observed that a 60% LE content (CLE 7, 5490%) leads to an increase in *Q_max_* to almost four times higher than seen with the CLE 1 sample (1460%). The release kinetics of PrHy indicate for all hydrogels a non-Fickian transport mechanism, where both the water diffusion rate and the relaxation rate of the polymer chains control the overall drug release process. The influence of the composition of CLE hydrogels can also be observed on the mechanical properties of the samples. The increase in the LE content in the structure of the hydrogels causes a decrease in the compression modulus, due to the fact that the presence of LE prevents the formation of cross-linking bridges between the cellulose chains, which results in the formation of softer and more porous hydrogels, suitable for being used in wound healing. Moreover, the values obtained for the mucoadhesion force of the CLE hydrogels show that the samples fall within the allowed range for the adhesion process of oral dressings, which recommends their use as oral wound dressings. Studies on the antimicrobial activity of CLE hydrogels against *Escherichia coli* and *Staphylococcus aureus* show that all the hydrogels are effective against these bacteria, starting from CLE 4, which demonstrates the real potential of these materials to be used as antibacterial dressings in medical treatments. An additional improvement in the antimicrobial properties of the CLE hydrogels was induced by the presence of PrHy in the P-CLE hydrogels. In vitro biocompatibility studies indicate that both CLE and P-CLE hydrogels have good viability and can be considered as non-cytotoxic materials. Furthermore, by adding LE into the composition of CLE hydrogels, an obvious improvement in cell viability is achieved. 

The entire set of characterization techniques used in the study have revealed that these new CLE hydrogels are biocompatible, have high swelling capacity and antibacterial properties, can release drugs in a controlled manner, and present mucoadhesiveness. Overall, the findings of the study highlight the fact that the CLE hydrogels can be used as possible oral dressings in wound management.

## Figures and Tables

**Table 1:** Composition and main characteristics of CLE hydrogels.

Samples	Hydrogels Composition		Hydrogels Features		
	Cellulose, %	LE, %	Gel Fraction Yield, %	*Q_eq_*, %	*Q_max_*, %
CLE 1	100	0	99.9	1920	1460
CLE 2	90	10	95.7	3250	1810
CLE 3	80	20	92.5	3550	2050
CLE 4	70	30	80.8	4010	3480
CLE 5	60	40	78.5	5750	4360
CLE 6	50	50	76.0	7310	4160
CLE 7	40	60	54.8	10,265	5490

**Table 2:** Swelling kinetic parameters for CLE hydrogels.

Samples	Swelling Kinetic Parameters		
	*n_sw_*	*k_sw_*	R^2^
CLE 1	0.080	0.636	0.994
CLE 2	0.140	0.672	0.996
CLE 3	0.147	0.818	0.997
CLE 4	0.201	0.853	0.996
CLE 5	0.255	0.889	0.999
CLE 6	0.265	0.936	0.994
CLE 7	0.352	0.943	0.999

**Table 3:** Cellulose infrared crystallinity ratios and hydrogen bond intensity.

Samples	IR Crystallinity Ratio		HBI	*E_H_*, kJ	Δ*H*, J/g	a/b
	TCI	LOI				
	A_1375_/A_2892_	A_1420_/A_893_	A_3400_/A_1320_			
CLE 1	0.55	0.96	5.28	4.57	126.60	0.92
CLE 2	0.53	1.21	4.59	4.54	125.37	0.92
CLE 3	0.52	1.23	4.49	4.52	125.78	0.92
CLE 4	0.53	1.36	4.36	4.37	121.65	0.92
CLE 5	0.51	1.48	4.08	4.35	121.23	0.89
CLE 6	0.51	1.52	4.01	4.33	120.82	0.85
CLE 7	0.48	2.06	3.96	4.30	119.99	0.81

**Table 4:** Absorbance ratios used for the evaluation of structural modifications in CLE hydrogels.

Samples	A_1266_/A_2892_	A_1106_/A_2892_	A_1060_/A_2892_	A_1508_/A_2892_	A_931_/A_2892_
CLE 1	0.66	4.13	6.65	-	1.08
CLE 2	0.59	3.79	6.07	0.06	0.83
CLE 3	0.41	2.58	4.06	0.12	0.53
CLE 4	0.31	1.89	3.06	0.13	0.53
CLE 5	0.28	1.98	2.36	0.18	0.49
CLE 6	0.27	1.61	2.50	0.19	0.47
CLE 7	0.23	1.52	2.32	0.22	0.25

**Table 5:** Values of yield stress, dynamic moduli at the crossover point (G′ = G″), and the stress and strain at the flow point (δf and γf).

Sample	Yield Stress, Pa	G′ = G″, Pa	δ_f_, Pa	γ_f_, %
CLE 1	118.0	-	-	-
CLE 4	19.0	631.9	569.7	62.5
CLE 7	8.5	159.5	115.6	51.1

**Table 6:** Degree of incorporation (Id) and kinetic parameters of PrHy released from P-CLE hydrogels.

Sample	Kinetic Parameters of PrHy Release			Id, %
	*n_r_*	*k_r_*	R^2^	
P-CLE 1	0.602	1.942	0.995	11.50
P-CLE 2	0.579	1.975	0.997	13.89
P-CLE 3	0.576	2.036	0.999	16.19
P-CLE 4	0.576	2.231	0.997	19.31
P-CLE 5	0.535	2.343	0.995	21.93
P-CLE 6	0.515	2.486	0.996	27.20
P-CLE 7	0.503	2.630	0.995	29.51

**Table 7:** Antibacterial activity of CLE and P-CLE hydrogels.

Sample	Growth Inhibition, %	
	*Escherichia coli*	*Staphylococcus aureus*
CLE 1	20	14
CLE 2	70	59
CLE 4	81	65
CLE 6	91	85
P-CLE 1	36	16
P-CLE 2	80	43
P-CLE 4	85	64
P-CLE 6	96	88

## Data Availability

Data are contained within the article.
